# Social computing for image matching

**DOI:** 10.1371/journal.pone.0197576

**Published:** 2018-05-29

**Authors:** Pablo Chamoso, Alberto Rivas, Ramiro Sánchez-Torres, Sara Rodríguez

**Affiliations:** BISITE Digital Innovation Hub, Edificio Multiusos I+D+i, University of Salamanca, 37007, Salamanca, Spain; IBM, UNITED STATES

## Abstract

One of the main technological trends in the last five years is mass data analysis. This trend is due in part to the emergence of concepts such as social networks, which generate a large volume of data that can provide added value through their analysis. This article is focused on a business and employment-oriented social network. More specifically, it focuses on the analysis of information provided by different users in image form. The images are analyzed to detect whether other existing users have posted or talked about the same image, even if the image has undergone some type of modification such as watermarks or color filters. This makes it possible to establish new connections among unknown users by detecting what they are posting or whether they are talking about the same images. The proposed solution consists of an image matching algorithm, which is based on the rapid calculation and comparison of hashes. However, there is a computationally expensive aspect in charge of revoking possible image transformations. As a result, the image matching process is supported by a distributed forecasting system that enables or disables nodes to serve all the possible requests. The proposed system has shown promising results for matching modified images, especially when compared with other existing systems.

## Introduction

The large amount of data available, mainly on the Internet, has made the analysis of such data one of the main trends of computational research in recent years [1]. It has been demonstrated that a correct analysis of this data can provide valuable information to those who perform the analysis in multiple areas.

The data may have different sources, which may be public or private, reliable or unreliable. More specifically, the sources providing or generating data in the case of social networks can be the users who are connected to the Internet and decide to share information.

This article focuses on the analysis of images published in social networks, more specifically a business and employment-oriented social network called beBee [2]. In this case, the intention is to contact users with the same interests based on their publications. The users provide images as data. As this data may or may not be accompanied by a descriptive text, the text itself will not be taken into account.

Therefore, the problem to be solved is to identify which images are the same from a human point of view, even though they are not computationally the same for one or several reasons: i) the quality has been modified or the image format has been changed; ii) a watermark has been included; iii) color filters have been applied; iv) a frame has been added or removed; v) the image has been rotated.

The analysis of these data supposes a series of challenges. The solution for the image matching presented in this article attempts to solve this problem with an algorithm based on obtaining features from the images as hashes, so that the system is able to quickly compare the existing images and the new image at the moment it is sent by the user. In order to solve the problem of massive data analysis, a forecasting system was designed to address the high volume of requests by modifying the cluster configuration.

The whole platform is supported by a social computing system. The proposed system uses a new open architecture based on the open concept of a virtual organization (VO) [3] of agents designed around the notion of social computing and able to emulate a human organizational model and behavior. The combination of these techniques provides advanced capabilities for adaptation and communication and is suitable for problems like the present one in which aspects such as social networks, distributed computing and social sense-making fit together.

The remainder of the article describes the existing methodologies regarding social computing, image matching, and forecasting mechanisms. The proposed system is described, including the algorithm for image processing and matching, as well as the platform that supports real-time processing. This system is evaluated in the results section with a set of images. Finally, the conclusions drawn and the lines of future work are presented.

## Background

As previously mentioned, the main focus of this article is the identification of images that are the same from a human point of view but different computationally, in order to connect users who publish similar images, given that they are likely to have common interests and new business opportunities may emerge.

When analyzing whether two images are the same, it is necessary to perform a series of checks due to the fact that two apparently identical images may be computationally different due to their compression, difference in quality, number of colors or size, slight modifications of the image with filters or watermarks, changes in the tonality, insertion of frames, or rotations.

There are different existing methodologies that attempt to provide a solution to a similar problem, but many of them do not cover all the possible transformations that can be made to image without appearing different to the human eye.

Another problem to solve is the large volume of images to compare when an image is uploaded by a user. This system has to be integrated in a public and international social network (beBee) with more than 10 million users who have no limitations when uploading content, which leads to the problems of large volume of data and the high processing capacity required. It is therefore necessary for the designed solution to be capable of applying mass data processing techniques to provide a solution in the shortest possible time so that users are not inconvenienced by the processing time.

This section details the different existing methodologies that have been successfully applied to similar problems, separating the social computing focus from image matching and the prediction of computational requirements.

### Social computing

The evolution of software and, more to the point, of the software that incorporates elements of artificial intelligence, tends towards the creation of entities with social behaviors and conduct similar to those of human beings. The theory of agents rests on the concept of agent [4]. An agent is an autonomous entity endowed with certain capacities typical of human beings. It may be seen as a development of the concept of software object, perfected as a result of the influence of artificial intelligence, which allows characteristics such as rationality, intelligence, autonomy and learning to be incorporated [5] [6]. As in the case of human beings, agents must have social skills and be able to perform tasks or solve problems in a distributed fashion. One then speaks in terms of a multi-agent system, in which the agents cooperate and interact to achieve the final aims of the system.

An idea that seems to be gaining considerable ground is that modeling the interactions of a multi-agent system cannot be related exclusively to the actual agent and its communication capabilities, but must involve the use of concepts found in social and organizational engineering as well [3] [7]. It is possible to establish different types of agent organizations according to the type of communication, the coordination among agents, and the type of agents that constitute the group. Each organization needs to be supported by a coordinated effort that explicitly determines how the agents should be organized and carry out the actions and tasks assigned to them [3] [7] [8].

In this sense, social computing is a general term for an area of computer science that is concerned with the intersection of social behavior and computational systems [9] [10]. Social computing is basically the use of computers for social purposes [11]. Before the Internet, computers were largely used as tools for increasing productivity. In a Social Computer, the Internet supports the infrastructures within which social interactions and problem-solving activities will be performed according to the deeply interactive norms and patterns that regulate societies. Key aspects include the experience of end users interacting with the Social Machine and the user perception [11] [10] [12].

Computers were largely used as tools for increasing productivity, however, the Internet introduced a social element that allows the users to collaborate, share interests, publish personal insights and use the computers in a social way. Some authors define Social Computing as the computational facilitation of social studies and human social dynamics as well as the design and use of new technologies that consider social context. For Robertson et al. [10] the power of the Social Computer resides in the programmable combination of contributions from both humans and computers:

- On the one hand, within organized social computation workflows, humans bring their competences, knowledge and skills, together with their networks of social relationships and their understanding of social structures.
- On the other hand, new technologies can search for and deliver relevant information. Humans can then use this information within their contexts to achieve their goals and, eventually, to improve the overall environment in which they live.

Social Computing has evolved during recent years to provide more realistic ways to improve social behaviors and relationships using computer science, such as argumentation techniques [13]. The existing solutions have focused on theoretical underprintings, technological infrastructure and applications [10]. These range from simple forms of social interaction to the coordination of large-scale collaborative efforts, and it is necessary to provide new solutions for: various forms of socially-distributed problem-solving; various aspects of social relationship management (including the formation; maintenance and dissolution of both professional and personal relationships); and various aspects of social cognition or social sense-making (for example, person perception, social networks or image processing). Thus, it is desirable to create social tools with the capacity for self-organization and self-adaptation, and intelligent agents and multi-agent systems are very appropriate for these purposes. Social computing is a key concept to design social applications, providing new ways for interaction with human societies. In this sense, the use of a VO of agents and social computing strategies with advanced capabilities for adaptation and communication is suitable for problems like the present one in which aspects such as social networks, distributed computing and social sense-making are conjugated [14] [15] [16] [17]. These techniques will make it possible to approach problems in a distributed fashion while designing agents specialized in particular tasks such as the integration of prediction technologies, communications and exchange of information, interaction with users, analysis of images published in social networks, etc.

In order to execute these tasks, the proposed system uses a new open architecture based on the open concept of VO [3] agents and based on a multiagent system architecture designed around the concept of social computing and the ability to emulate human organizational model and behavior. A system like this will be able to create virtual organizations of agents that will resemble the organizations present in human societies that exist in a controlled environment. The system will then be able to manage information and offer services to the users, considering social and organizational behaviors as well as the available data in order to provide a management paradigm close to reality.

### Image matching

In the fields of computer vision and image processing, different methodologies have been presented to extract relevant information with an image as input. These techniques are cataloged under the concept of feature detection.

There are different types of image features like edges, corners or interest points, and blobs or regions of interest. In this sense, there are multiple algorithms used to process images in search of features, the most common of which are:

- **Edges**: Canny, Sobel, Harris & Stephens, SUSAN [18].
- **Corners and points of interest**: Harris & Stephens, SUSAN, Shi & Tomasi, Level curve curvature, FAST [19], Laplacian of Gaussian, Difference of Gaussians, Determinant of Hessian, SURF [20], ORB [21].
- **Blobs**: FAST, Laplacian of Gaussian, Difference of Gaussians, Determinant of Hessian, Maximally stable extremal regions (MSER) [22], Principal curvature-based region detector (PCBR) [23], Gray-level blobs.

However, image classification can also be done in different ways, from object recognition and bag of word classifications to disregarding the image content entirely and using strict cryptographic hashes.

Perceptual hashing is a concept similar to the classical paradigm of cryptographic hashes, where the smallest change avalanches into an entirely different hash. In perceptual hashing the images content is used in an attempt to fingerprint the image, so that even if hashes are not identical they can be used to determine how “close” the images are to one another. Moreover hash based algorithms also have an amount of ductility when it comes to changes, so changing a single pixel for example will not change the generated hash in most cases.

Another important concept that has been applied when making comparisons between different images is the Hamming distance [24], which can be used on most of the resulting hashes to obtain the perceived difference between two images in that a perceptually similar image would have a short hamming distance, 0 for the same image. A quick definition for hamming distance, *d*(*x*, *y*), is the number of ways in which *x* and *y* differ. In other words, the hamming distance is simply the number of positions in which they are different. It is calculated as shown in Eq (1).

$$\begin{array}{c} D_{H}=\sum_{i=1}^{k}\left|x_{i}-y_{i}\right| \end{array}$$ (1)

**Fig 1:**
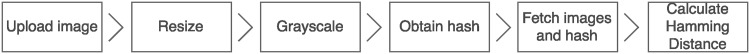
Typical hash-based algorithms flow diagram.

There are different proposed algorithms based on the hash value generation technique: pHash (also called “Perceptive Hash”, with different variations) [25], aHash (also called Average Hash or Mean Hash) and dHash Also called Difference Hash) [26]. The typical hash-based algorithms flow diagram is shown in Fig 1.

However, all variations of this methodology present different problems when dealing with an image that has been rotated or to which a frame has been added, for example.

For the former problem, a modification of these steps is proposed in [27] by introducing a rotational system whereby it is possible to differentiate images rotated 22.5º; however, this implies a loss of precision in the corners when the original images are rectangular or square, the most common shape of images uploaded to social networks, so its solution is not applicable to the present problem.

There are on-line platforms dedicated to the search of images existing on the Internet similar to one provided by the user, without taking into account the meta-data or associated text. Their applicability is oriented to the search of image plagiarism. This is the case of TinEye [28], whose algorithm is not public, but it is based on the analysis of hashes.

### Forecasting systems

The volume of traffic that a public and international social network can generate is so high that it is necessary to apply methodologies that can manage all the requests to be able to provide a solution in a short period of time; so short in fact, that it is practically real time. This problem can be addressed by adapting the number of nodes in charge of attending the received image matching requests so that all of that requests can be answered in less than two seconds. However, these nodes have a booting period (*bp*) of five minutes, which makes it impossible to decide the number of nodes needed to attend the current requests in real time. For this reason, it is necessary to estimate the number of nodes required to serve all the coming requests during the following *t* + *bp*, *t* + 2*bp*.

Prediction systems can be classified as either qualitative or quantitative.

Qualitative systems are based on different techniques which require human expert participation to obtain conclusions, such as forecasting based on the Delphi method. To address our specific problem, the system must be readapted every certain amount of time without human interaction. As a result, these kinds of forecasting systems are not applicable.

Quantitative systems are in this case the best option to use. In general terms they can be classified as:

- **Temporal series forecasting**: It is based on the patterns in the evolution of a variable, usually univariant [29].
  - Advantages: It is easy to implement these methods and they need few historical data.
  - Disadvantages: Their precision is dependent on the complexity in the behavior of the forecasted variable. If it depends on multiple variables then these methods cannot predict changes well, as they do not adapt well to causality.
  - Conclusion: Our number of requests depends on the day, number of users and holidays; therefore, one option could be to segment the historical data to apply the method selected for each one. The problem is that these methods also need a lot of time to make a forecast for each value, so we have discarded them in favor of a faster and more flexible method.
- **Probability forecasting**: Based on calculating a range of probabilities for different outcomes in the prediction, usually according to probability distributions. In string theory, arrivals are usually assumed to be Poisson distributed because of their memoryless characteristic [30]. Another commonly used option is Bayesian networks [31].
  - Advantages: We can calculate the probability of each forecasted value and also cumulative probabilities. Moreover, these methods are usually fast to compute and do not need large amounts of data.
  - Disadvantages: Methods based on distributions, like Poisson, cannot learn patterns from historical data.
  - Conclusion: We consider Poisson distribution to be more useful in this case than other methods because we assume requests follow this distribution and also because our need is to fulfill the restriction, so we focus on the security of not surpassing the two second limit over the savings. In this regard, Poisson distribution is effective because it allows us to calculate cumulative probabilities.
- **Causal forecasting**: These methods are multivariant, focusing on modeling the causality and correlation in the historical data to forecast.
  - Machine Learning: Most common methods used in machine learning are: Bayesian networks [31], SVR (support vector regression) [32] and ANN (Artificial Neural Network) [33].
  - Other methods: Although there are multiple causal forecasting methods, our objective is for the system to learn from past data automatically, so we continue with machine learning.
    ∗ Advantages: Machine learning allows us to detect patterns in the historical data but also to take into account multiple variables, allowing the forecast to adapt to changes. They are usually fast at the moment of prediction.
    ∗ Disadvantages: Training machine learning algorithms is usually a slow process. They need large amounts of data to detect the right patterns and make useful forecasts.
    ∗ Conclusion: We have used SVR for the forecast because we believe it is better suited to the problem than other methods.

In conclusion, we discarded temporal series for several reasons: in our current problem we need to detect patterns in the historical data; temporal series are computationally slower than machine learning at the moment of making a forecast; and we need to take into account multiple variables such as the number of registered users. As a result, we chose SVR, a machine learning method. However, it is not without its problems: SVR cannot adapt well to changes that do not follow historical patterns and needs a larger amount of historical data to forecast correctly. Therefore, a probabilistic method based on Poisson distribution with a moving window fits our solution. We call this two parts static and two parts dynamic. We will explain how this hybrid system works in a subsequent section.

The static part is responsible for adjusting to unforeseeable changes from the analysis of historical data, complementing the static part. The probability of events, in our current case the number of image uploads at a certain interval, can be estimated by applying a Poisson distribution, as defined by Eq (2). This is because, according to queuing theory, it is possible to assume a distribution of requests governed by a Poisson distribution.

$$\begin{array}{c} P\left(k,\lambda \right)=\frac{e^{-\lambda }\lambda^{k}}{k!}=P\left(X=x\right) \end{array}$$ (2)

where *λ* (λ∈R,λ>0), is the average number of image uploads per interval, *e* is the Euler’s number and *k* is the number of image uploads. The result is the probability of receiving *k* (k∈N∪{0}) image uploads.

### Conclusion

Once the state of the art is realized, social computing is able to provide a suitable model to manage the desired solution. This solution must be based on an image matching system. The best one to face the proposed problem are hash based systems because comparison is based on Hamming distances, which can be easily implemented with just one OR operation, which is very quick. However, the number of requests has to be estimated in order to deploy the nodes needed to serve those requests, and hybrid forecasting systems are the best model to use.

## Proposed system

**Fig 2:**
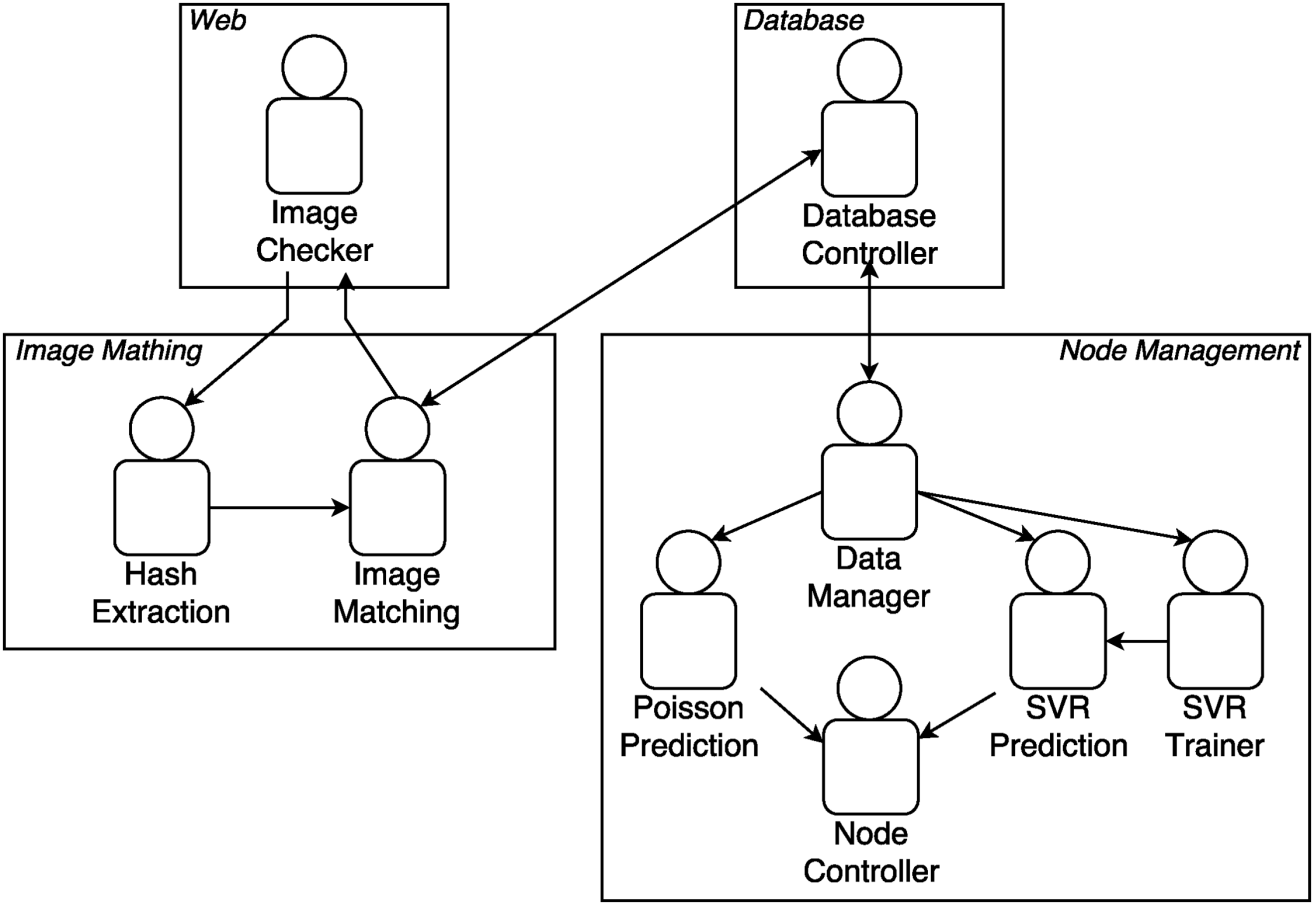
Multi-agent system overview.

The proposed methodology is based on Virtual Organizations (VOs) of agents that contains different roles that agents can implement to solve more specific problems. As shown in Fig 2, there are four VOs, and the entire system contains nine roles that can be implemented by one or more agents, depending on the case.

The most important VOs are *Image Matching* and *Node Management*. As described above, image matching is associated to the computational process in order to find the same image, even if images have been slightly modified. In order to match the images, the system implements an algorithm for every new input image, so the computational requirements can be really high. A solution to provide all the needed computational capacity without wasting resources is implemented in the *Node Management* VO.

The proposed system is a separate solution from the social network, which is used through web services so that the computational component for the image matching system is completely decoupled from the social network computational component.

With more than 10 million users in social networks, the new system must be able to provide enough computational processing to serve all the requests that are generated when any user uploads a new image.

For the social network, user friendliness is a very important issue. For this reason, all requests must be processed in less than two seconds. This processing time depends directly on the number of received requests as well as the number and the capacity of nodes (all the used nodes have the same characteristics) that are responding to those requests. The system must be able to forecast the number of predictions to determine the number of nodes to deploy. As nodes have a booting period of five minutes, the predicted number of requests belongs to the interval associated from the following five minutes to the following ten minutes.

When designing a forecasting system it is really important to consider both the users’ habits and any unforeseen changes (such as the celebration of certain events). Our proposal is, therefore, based on a hybrid forecasting system composed of two parts: i) a static and ii) a dynamic component.

- The static component is in charge of recognizing historical request patterns and forecasting the number of future predictions to deploy the number of required nodes that guarantee the requests are answered in less than two seconds. The system proposed to address this static component is based on a SVR, which recognizes patterns with the following inputs: daytime, weekday, public holiday or not, month, number of active users, current value, previous value.
- The dynamic component is in charge of readjusting the system in case of an increase in the number of predictions, not detectable by the static component. In this case, the proposal uses the Poisson distribution because we can assume that requests follow this distribution because of the queueing theory basis. Therefore, different equations based on Poisson distribution are applied, allowing the number of requests to be forecasted within a confidence interval. These are the equations that describe this part of the system:
  $$\begin{array}{c} CDF=P\left(X\leq k\right)=e^{-\lambda }\sum_{i=1}^{\left\lfloor k\right\rfloor }\frac{\lambda^{i}}{i!}=\sum_{i=0}^{\left\lfloor k\right\rfloor }P\left(k,\lambda \right)=P_{AC}\left(k,\lambda \right)=prob \end{array}$$ (3)
  where *prob* ∈ [0, 1]
  Eq (3) is the cumulative probability up to a number of requests *k*.
  $$\begin{array}{c} CDF^{-1}=max\left\{k\in N\cup \left\{0\right\}:P_{AC}\left(k,\lambda \right)\leq prob\right\}=P_{AC}^{-1}\left(prob,\lambda \right) \end{array}$$ (4)
  Eq (4) is the quantile function for the Poisson distribution, or inverse cumulative probability, which is giving the number of requests up to the cumulative probability as a percentage. We will use it to calculate the number of requests to which demand is likely to evolve based on a confidence interval (it will be the percentage sent to the function).
  $$\begin{array}{c} CDF^{-1}=k=X_{U}=P^{-1}\left(X\leq k\right) \end{array}$$ (5)
  $$\begin{array}{c} probError=1-prob \end{array}$$ (6)
  The *probError* represented in Eq (6) is based on the margin of confidence.
  $$\begin{array}{c} prob=1-probError \end{array}$$ (7)
  *prob* is the confidence margin, as shown in Eq (7).
  $$\begin{array}{c} RPC=rpn-(X_{U}-\left(X_{U}\;\mathrm{mod}\;rpn\right)rpn) \end{array}$$ (8)
  where *rpn* (requests per node) is the maximum number of requests that a node can manage to process in less than two seconds.
  *RPC* (Remaining Processing Capacity), defined in Eq (8), is the margin from the number of predicted request *X*_*U*_ up to the number of requests that can be handled by the open nodes.
  $$\begin{array}{c} LIM_{U}=CDF^{-1}\left(prob,\lambda \right)+RPC-\lambda \end{array}$$ (9)
  *LIM*_*U*_, represented in Eq (9), is the increase in the number of requests that can be handled by the nodes predicted by the dynamic system.
  $$\begin{array}{c} GR(t)=\{x:x=req(t)-req(t-1)\} \end{array}$$ (10)
  *GR*(*t*) in Eq (10), is the request growth rate.
  $$\begin{array}{c} GR_{+1}\left(t\right)=\left\{x:x=req\left(t\right)-req\left(t-2\right)\right\} \end{array}$$ (11)
  *GR*_+1_(*t*) is the request growth rate calculated with *t* − 2 to be able to calculate the limit of growth that supports the dynamic system, it is represented in Eq (11).
  $$\begin{array}{c} Ov\left(x,y\right)=\sum_{t=x}^{y}\left|LIM_{U}\left(t\right)-GR\left(t\right)\right| \end{array}$$ (12)
  *Ov*(*x*, *y*), defined in Eq (12), is the system overload between two instants of time *x* and *y*, that is, the increase in the number of requests that exceeds the capacity of the dynamic system with a confidence interval *prob* fixed.

After both systems are combined, the number of nodes that are finally considered is the higher forecast. This option promotes the processing guarantee at the expense of saving in computational cost.

**Fig 3:**
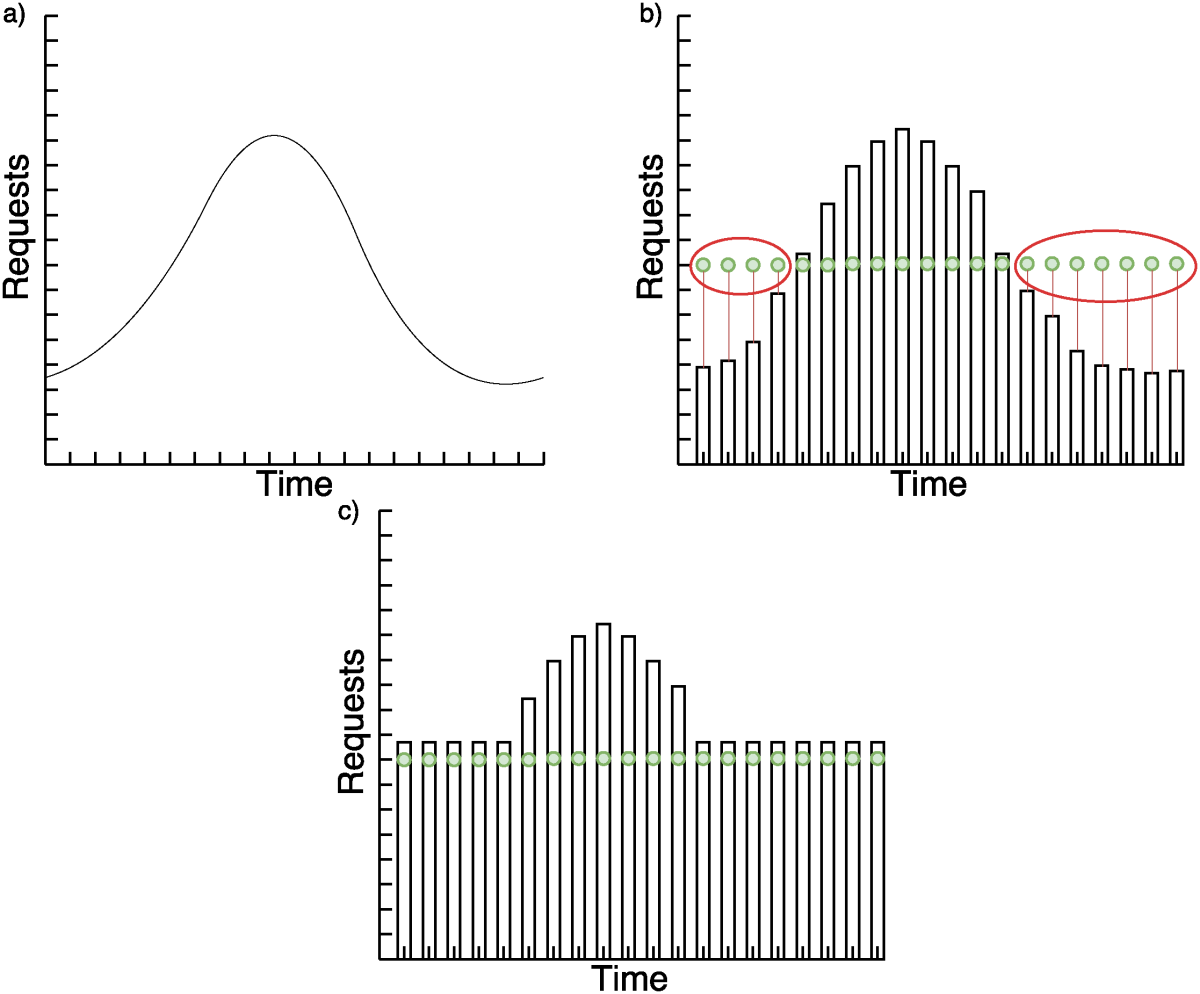
a) Abstract representation of requests pattern based on historical data; b) Static system problem with unexpected continuous requests; c) Hybrid forecasting system with unexpected continuous requests.

**Fig 4:**
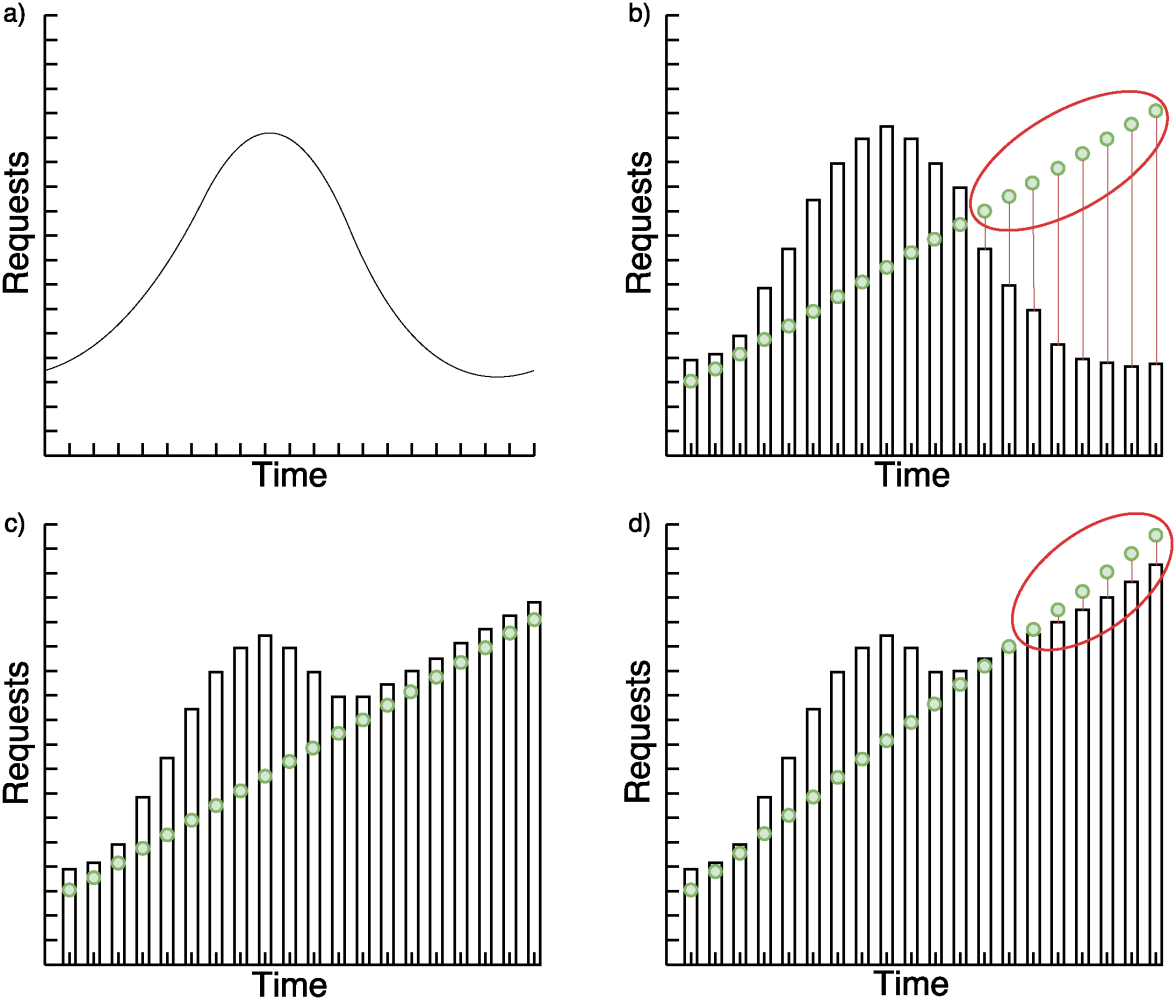
a) Expected requests pattern based on historical data; b) Static system problem with unexpected increasing requests; c) Hybrid forecasting system with unexpected increasing requests; d) Hybrid forecasting system with unexpected increasing requests over the confidence interval.

**Fig 5:**
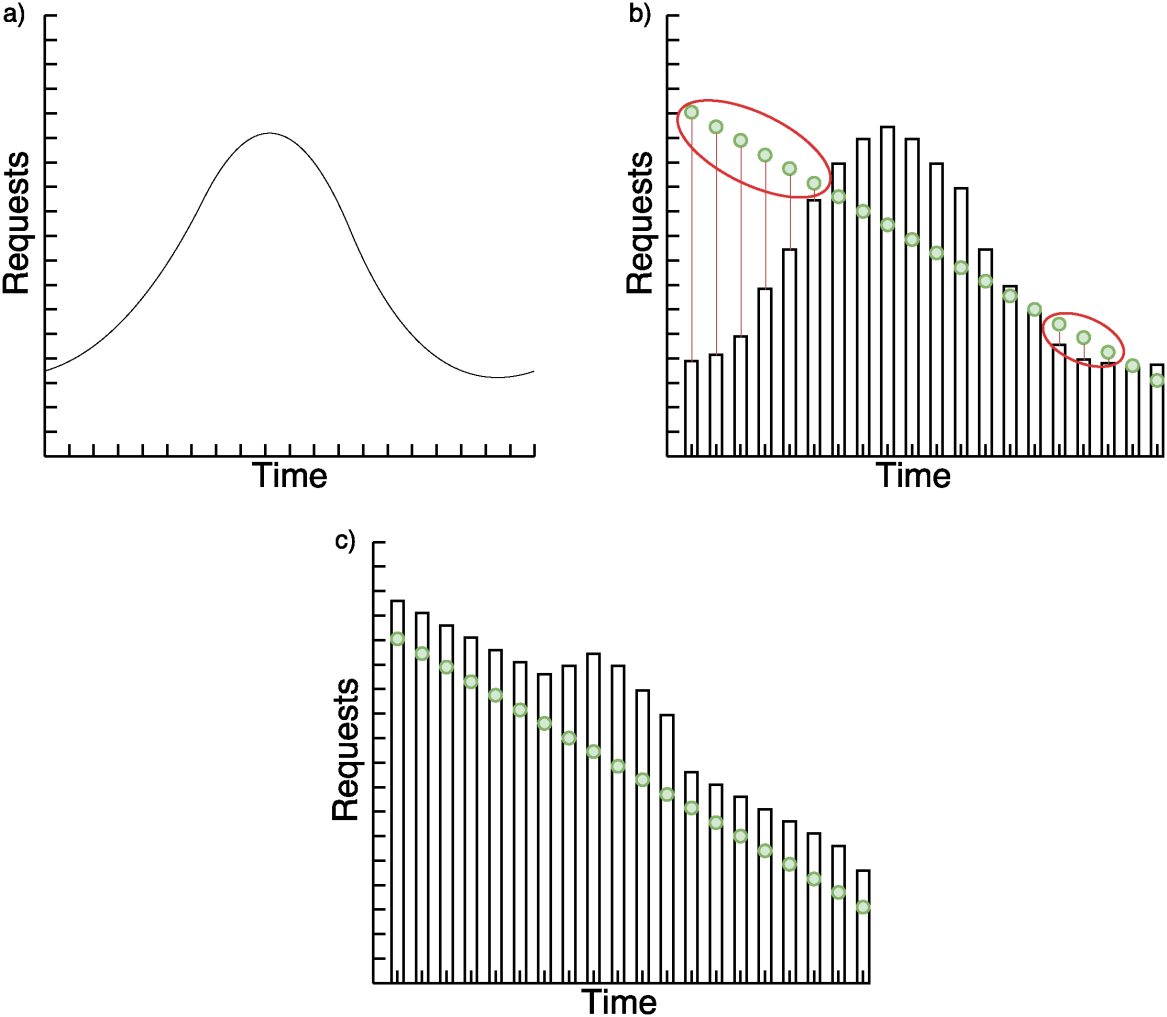
a) Expected requests pattern based on historical data; b) Static system problem with unexpected decreasing requests; c) Hybrid forecasting system with unexpected decreasing requests.

We analyze three base cases of requests behavior to explain the hybrid system in Figs 3–5. In the images, the green points to which a time tag is attached, represent the number of requests received in a period of time. Bars represent the number of requests that nodes forecasted can satisfy in less than two seconds.

In Fig 3 we can see that the SVR failed to recognize a constant number of requests when the pattern it had learned is different. This is solved with a dynamic system.

Fig 4 shows the same problem as the continuous request case with the SVR. A dynamic system solves it but only when the rate of growth seen in Eq (11) is less than the limit from Eq (9). This can be adjusted with the probability of the confidence interval in Eq (7).

In Fig 5 we can see the same problem as the continuous and ascending request cases with the SVR. Dynamic system solves the problem by adding extra costs because it opens more nodes than necessary. This too can be adjusted with the probability of the confidence interval in Eq (7), but in our particular case we prefer the security of complying with the restriction than the savings.

Every part of the system can now be easily understood. The system structure is detailed below from a VO approach.

### Web virtual organization

This VO contains only one role, **Image Checker**, implemented by just one agent, who is in charge of receiving requests from the website through a RESTful web service and returning the most similar image(s) in JSON format. To do so, this agent establishes communication with the agents included in the Image Matching VO.

In addition, whenever a valid request is received, it is communicated to the Data Manager included in the Node Management VO to take into account the real number of petitions.

### Image matching virtual organization

Two roles can be found in this VO: Hash Extraction and Image Matching. The former is implemented by agents in charge of obtaining the hash value for every uploaded image, while the latter is implemented by agents in charge of matching that hash with existing hashes by calculating the Hamming distance.

#### Hash extraction role

One of the main keys of the proposed system, which improves the result of similar systems, is the preprocessing stage. This stage is focused on applying a series of transformations to the images that are received as input by the user. This is followed by a scheme similar to hash-based algorithms.

Hash-based algorithms are best adapted to our image matching problem because of their speed. The pHash approach extends the aHash approach to the extreme, using a Discrete Cosine Transform (DCT) [34] to reduce the frequencies. We have followed a similar schema, but the algorithm presents important improvements.

The possible transformations that a user can perform on an image, after which the system will still considered it to be the same image, are: i) insertion of an outer uniform frame; ii) rotation of the image; iii) insertion of a watermark. It should be noted that all hash-based algorithms are really robust to uniform transformations of the tonality, so this transformation has not been considered for the comparison, although the system tolerates such modifications.

When a watermark is inserted, a hash-based algorithm application can be sufficient to determine whether it is the same image despite the modification. Therefore, in this first stage of preprocessing, the proposed system focuses only on modifications based on the insertion of an outer uniform frame and also on the rotation of the image.

- **Solid frame addition**: The proposed system applies the Algorithm 1 so that the following steps of the methodology are always performed without considering the uniform outer frame. The first step is to transform the original image provided by the user of the social network *I* to a grayscale image *gI*, which will also be used in the following steps.
- **Image rotation**: The most common rotations that a user applies to an image are based on 90° modifications. It is in this type of rotations where this feature of the preprocessing is centered. The objective is for the images to follow a rotation pattern so that they always have the same orientation in the system. There are different possibilities that depend on the dimensions of the image, and the ability to find rectangular or square images.
  If the image is rectangular, the system will always work with the image in landscape mode (the two longest sides are in the x-axis). The system must then determine which side is placed on the top and which one is placed on the bottom. If the image is square, the previous logic can not be applied, since the four sides are the same length. In both cases, the key of the final orientation will be the tonality of the image, as described by Algorithm 2. Although this step appears in the preprocessing section, it is applied in an intermediate step of Algorithm 3, which will be detailed below, to avoid possible changes in the tonality resulting from the insertion of a watermark.

**Algorithm 1** Solid frame removal algorithm

1: **function** frameRemoval (*I*)

2:  *gI*′ = grayscale(*I*)

3:  **if** hasFrame(*gI*′) **then**    ▹ Check frame

4:   *value* = getFrameTonality(*gI*)    ▹ Get frame tonality value

5:   *bI* = toBinary(*gI*, *value*)    ▹ Frame tonality as threshold

6:   *cnt* = findContour(*bI*)    ▹ Get contour

7:   〈*x*, *y*, *width*, *height*〉 = boundingRect(*cnt*)    ▹ Find bounding rectangle

8:   *gI* = *gI*[*x*: *x* + *width*, *y*: *y* + *height*]    ▹ Crop grayscale image

9:  **else**

10:   *gI* = *gI*′

11:  **end if**

12:  **return** *gI*

13: **end function**

**Algorithm 2** Image rotation algorithm

1: **function** imageRotation (*gI*, *sI*)

2:  **〈** *width*, *height*〉 = getSize(*gI*)

3:  **if** *width* == *height* **then**    ▹ Square image

4:   *nsI* = *sI*[0: *width*, 0: *height*/2]    ▹ Get North middle

5:   *ssI* = *sI*[0: *width*, *height*/2: *height*]    ▹ Get South middle

6:   *wsI* = *sI*[0: *width*/2, 0: *height*]    ▹ Get West middle

7:   *esI* = *sI*[*width*/2: *width*, *height*/2: *height*]    ▹ Get East middle

8:   *highestMean* = getHighestValue($\bar{nsI},\bar{ssI},\bar{wsI},\bar{esI}$)

9:   **if** *highestMean* == $\bar{nsI}$ **then**    ▹ Highest tonality on top

10:    *sI* = rotate(*sI*, 180)

11:   **else if** *highestMean* == $\bar{wgI}$ **then**

12:    *sI* = rotate(*sI*, 270)

13:   **else if** *highestMean* == $\bar{egI}$ **then**

14:    *sI* = rotate(*sI*, 90)

15:   **end if**

16:  **else**    ▹ Rectangular image

17:   **if** *width* < *height* **then**

18:    *gI* = rotate(*sI*, 90)    ▹ Longest image side over x-axis

19:   **end if**

20:   *nsI* = *sI*[0 : *width*, 0 : *height*/2]    ▹ Get North middle

21:   *ssI* = *sI*[0 : *width*, *height*/2 : *height*]    ▹ Get South middle

22:   **if** $\bar{ngI}<\bar{sgI}$ **then**    ▹ Highest tonality on top

23:    *sI* = rotate(*sI*, 180)

24:   **end if**

25:  **end if**

26:  **return** *sI*

27: **end function**

The algorithm followed to obtain the hash associated with image I, provided by the user of the social network, is defined in Algorithm 3. Its input is the grayscale image *gI*, obtained in Algorithm 1.

**Algorithm 3** pHash-based algorithm

1: **function** getImageHash (*gI*)

2:  *sI* = rededuceSize(*gI*, 32, 32)    ▹ Reduce size to 32x32 pixels

3:  *rI* = imageRotation(*gI*, *sI*)    ▹ Rotate as defined in Algorithm 2

4:  *DCT* = computeDCT(*rI*, 32, 32)    ▹ Get a collection of frequencies and scalars

5:  *sDCT* = reduceDCT(*DCT*, 12, 12)    ▹ Get the lowest freq. (top-left 12x12)

6:  **for each** *px* ∈ *sDCT* **do**

7:   **if** $px>\bar{sDCT}$ **then**    ▹ Compare every pixel with sDCT mean

8:    *hash* = *hash* + 1

9:   **else**

10:    *hash* = *hash* + 0

11:   **end if**

12:  **end for**

13:  **return** *hash*

14: **end function**

The compute DCT function applies the one-dimensional DCT equation, which has been extended to apply to two-dimensional image arrays as described in Eq (13), by using matrix operations, which are much more efficient than a double nested for loop.

$$\begin{array}{c} DCT\left(i,j\right)=\frac{1}{\sqrt{2N}}C\left(i\right)C\left(j\right)\sum_{x=0}^{N-1}\sum_{y=0}^{N-1}px\left(x,y\right)\cos\left(\frac{2x+1}{2N}i\pi \right)\cos\left(\frac{2y+1}{2N}j\pi \right) \end{array}$$ (13)

where N is dimension of the input square matrix, 32 in this case, and the function *C*(*x*) is defined as described in Eq (14).

The formula yields an NxN square matrix of frequency coefficients. Each element of the output matrix is a coefficient by which the waveform of the corresponding spatial frequency is multiplied in the decomposition of the image sample.

$$\begin{array}{c} C\left(x\right)=\left\{ \begin{array}{cc} \frac{1}{\sqrt{2}} & \;\text{if}\;x=0\\ \text{1} & \;\text{if}\;x>0 \end{array}\right. \end{array}$$ (14)

Top-left 12x12 values are obtained because they represent the lowest frequency range. In contrast, the bottom right is the highest frequency range. The human eye is not very sensitive to high frequencies. Then, the result is reduced to a 12x12 matrix. As a result every image has the value of its associated hash, composed of 144 values (12x12) with value 1 or 0.

#### Image matching role

Once the hash of a new image has been extracted, an agent implements the Image Matching role and the Hamming distance is calculated by as defined in Eq (1). This is a quick operation that simply compares each bit position and counts the number of differences. Speed is a very important factor because of the huge size of the existing database, which is working over a document-oriented database. Using this kind of database makes it possible to group all similar image hashes into a single document and reduce the number of Hamming distances to calculate.

If the image is related to any of the existing images, the system must return as a solution the image with the greatest similarity found, since it will be the same image, whether modified or not. If the similarity level is 99% (the image has been modified for example with a watermark), it is stored in the database together with the similar image to reduce the processing time of future queries.

If the system cannot find any image whose similarity level is ≥99%, the hash of that image is stored in the database (the file of every image is stored in the social network servers, whether it is new or not).

### Database virtual organization

There is only one role, **Database Controller**. It is implemented by agents in charge receiving requests from Image Matching agents and translating them by applying BK-Trees [35]. This results in quicker database queries because the number of comparisons is reduced.

It also receives requests from the Data Manager agent to obtain all the required data to estimate the number of nodes to deploy, such as the current number or image uploads, or historical data.

### Node management virtual organization

This VO is responsible for determining the number of nodes that will be needed in the next period of time to provide service to the number of image matching requests that are estimated to have, and thus fulfill, the maximum processing restriction of two seconds.

**Fig 6:**
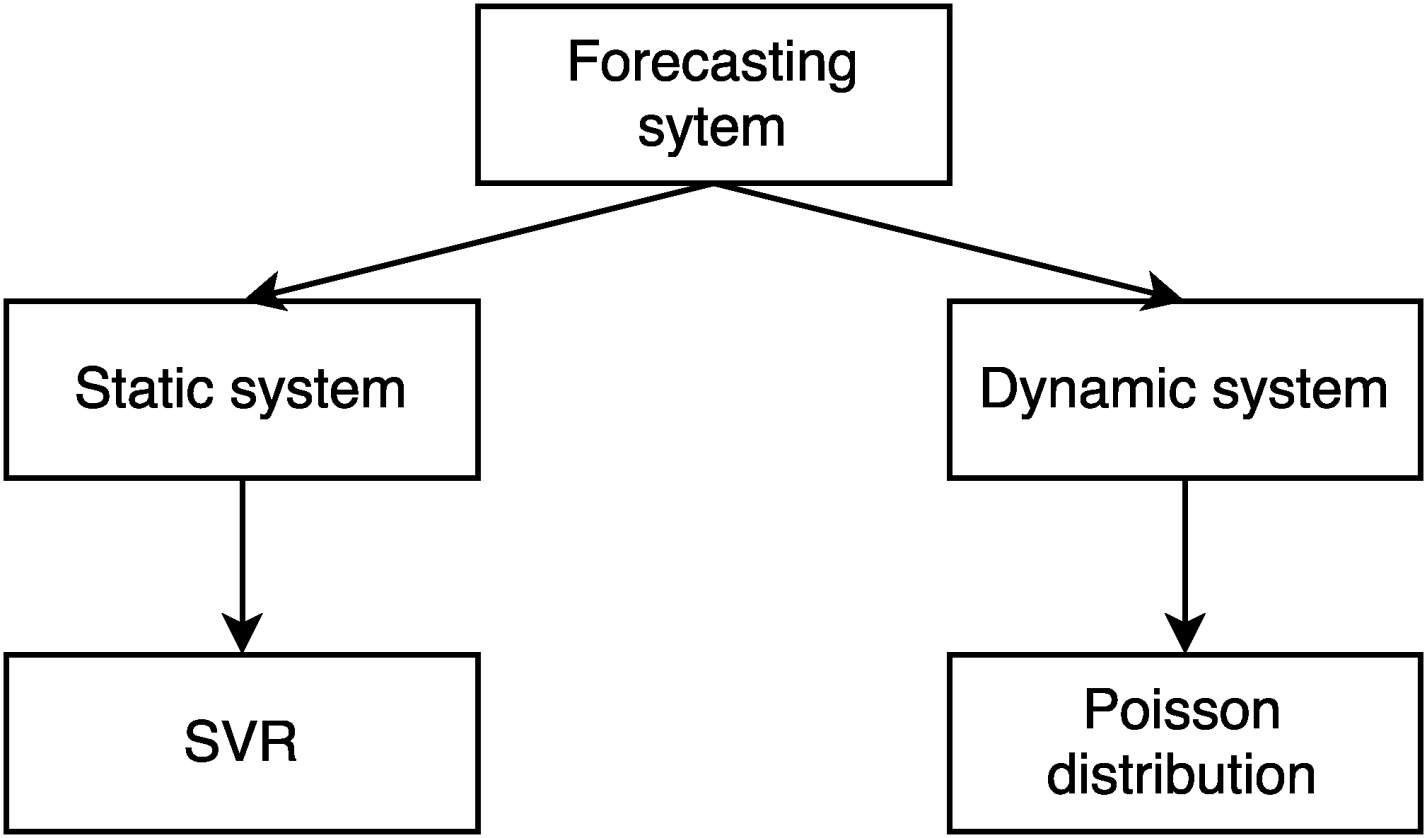
Node management—Forecasting system schema.

The VO is based on the previously explained division between dynamic system and static system, as shown in Fig 6.

The static system learns patterns based on historical data so that, although it may take into account increases in abrupt requests at certain times, such as holidays, it is not able to take into account unforeseen increases in requests, which may happen if there is an important event where they decide to use our system to upload the images.

The dynamic system solves this static problem by setting the open nodes to unpredictable increases based on the historical data. Its disadvantage is that it has a maximum adjustment of the growth rate based on the confidence interval. As a result, it needs to be adjusted to a balanced level between cost and security so that it fits adequately to unforeseen changes but does not keep too many Nodes open over time. This assumes that if the requests increase is higher than the dynamic system predicted with a confidence interval, there will be a period during which the requests will be processed in more than two seconds, not fulfilling the restriction until the system is readjusted. The system is readjusted as soon as the number of requests is again below the dynamic system setting. This problem is solved by the static system as long as the changes are learned through historical data. If they are not predictable, a higher confidence interval could also be set, which would increase the system’s ability to handle increased requests, but also increase the system cost.

The goals of the VO are mainly to achieve parallelism in the prediction, to increase the speed of the prediction system, and to have the different roles divided into agents to be able to expand the system flexibly.

It is subdivided into five roles that are explained below.

#### Data manager role

This role is implemented by one agent in charge of getting useful information so that prediction systems can determine the number of nodes required to support the system.

It receives information about all the image processing requests from the Database Controller. Although real requests are a continuous function, we use discrete values because the Database Controller stores the number of requests in one second intervals. It is also communicated with the Database Controller in order to manage the required persistent information.

Every time *n* ∗ *bp* (where *n* = 1, 2, … and *bp* is the node booting period), the number of nodes to deploy must be calculated. This agent starts the forecasting system to determine the number of the period from *n* ∗ *bp* + *bp* to *n* ∗ *bp* + 2 ∗ *bp*. To do this, the agent indicates the required information to the Poisson Prediction and the SVR agents, and they will in turn forecast the number of nodes concurrently.

Finally, the Data Manager agent is also communicated with the SVR trainer agent to send the required information to train the system.

#### SVR prediction role

Part of the static prediction is implemented with a SVR, which forecasts the number of predictions (which directly provides the number of nodes) by taking into account previous patterns which are mostly related to user habits.

The used SVR has the following inputs:

- Daytime: the daytime in minutes.
- Weekday: the current weekday (Monday, Tuesday, etc.).
- Month: the current month.
- Public holiday: a binary value which indicates whether the current day is a public holiday or not.
- Number of the social network active users.
- Request number in the time, *t*.
- Request number in the previous time, *t* − 1.

#### SVR trainer role

This role is implemented by one agent in charge of the SVR training. The objective when separating the SVR training from the SVR Prediction agent is to parallelize both processes so the whole system becomes faster. Once every training finishes, this agent updates the SVR Prediction agent with the new configuration.

#### Poisson prediction role

This role is implemented by one agent in charge of the dynamic forecasting aspect. It starts with the Data Manager agent requesting and using the received information.

**Fig 7:**
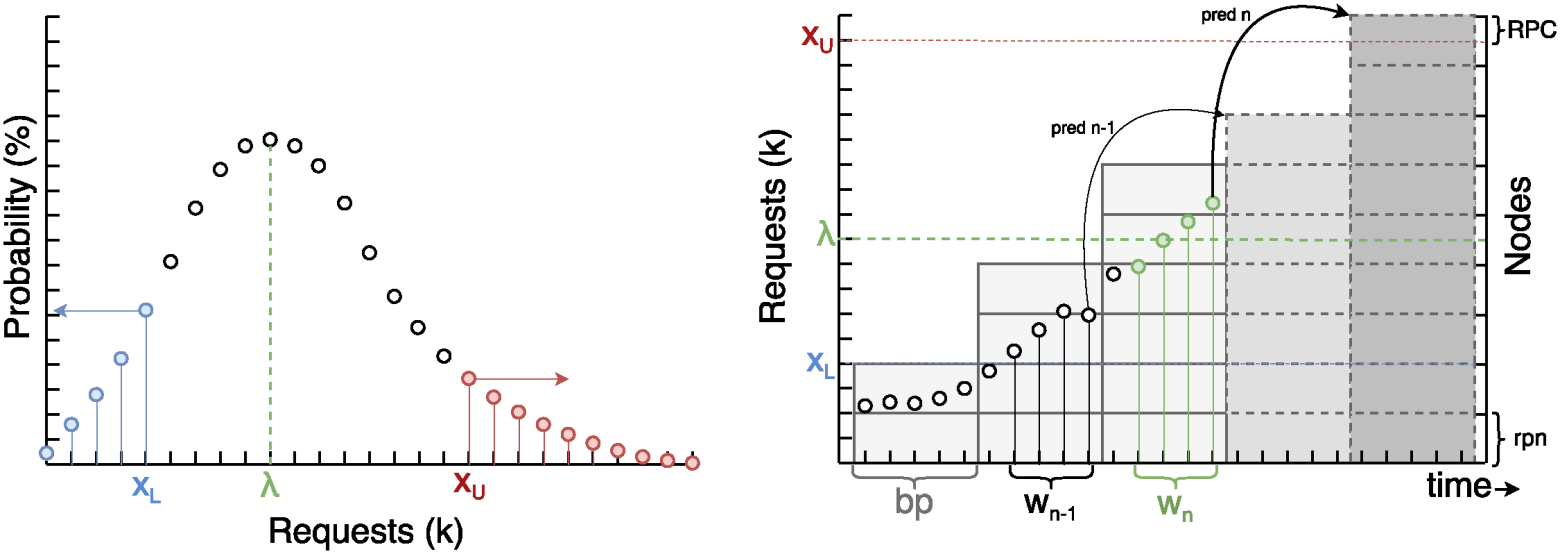
Dynamic forecasting behavior.

In Fig 7 we can see the Poisson probability distribution on the left and the forecasting based on that distribution on the right. In the Poisson probability distribution plot, every point represents the probability of occurrence of a specific number of requests in a discrete fashion. As shown in Eq (2) the Poisson distribution depends on *λ*, which is calculated with the requested values in the window of time *w*_*n*_. Then *X*_*U*_ is calculated with Eq (5) given a confidence interval, or the error as shown in Eq (7). *X*_*U*_ is the predicted maximum number of requests with the confidence interval given. In the plot on the right, every point represents the number of requests at a time in the past as a time series of historical data until the current moment. *bp* is the booting period which represents the interval of time needed to open a node. It is important to note that the prediction in a time *t* is for *t* + *bp*. The solid line rectangles represent the number of nodes opened, and when represented with dashed lines indicate the predicted nodes that depends on the *X*_*U*_ values predicted. *X*_*L*_ is the predicted minimum number of requests with the given probability.

Then, the inverse cumulative probability is calculated up to a selected confidence interval (in this case the selected value is 95%), the number of requests whose probability is under that value is considered the number of requests that the system will receive.

Once this value is obtained, it is sent to the Node Controller agent, which will determine the number of nodes that the system needs.

#### Node controller role

This role is implemented by one agent that determines the number of nodes that must be deployed to serve all the received requests. To determine this number, the forecasts obtained by the SVR Prediction and the Poisson Prediction agents are used. Since the priority of the system is to guarantee that all the requests are answered in less than two seconds, the number of nodes to deploy is defined by Eq (15)

$$\begin{array}{c} n=\lceil \frac{\max(req_{R},req_{P})}{rpn}\rceil \end{array}$$ (15)

where *n* is the number of required nodes at the instant *t* + *bp*, *req*_*R*_ is the number of requests forecasted by the SVR Prediction agent, *n*_*P*_ is the number of requests predicted by the Poisson Prediction agent, and *requestsPerNode* is the maximum number of requests that a node can process by guaranteeing that the answer is sent in less than two seconds.

## Results

The image matching software system has been developed in Java and its architecture is based on RESTful web services. The selected database is MongoDB (images are not stored in the DB) and a modified version of DC/OS which includes our forecasting system, is used to manage the nodes.

To perform the tests of the proposed system, a set of 200,000 images obtained from beBee were used as the image dataset. 1,000 of these images were chosen by searching existing images from the social network on the Internet. So, images obtained from the public image repository Pixabay [36] have been selected and we complied with the Pixabay terms of service. Only those with results in TinEye were selected, so we can compare the results with the TinEye algorithm. This dataset is published in [37].

Then, a script was used to apply the different transformations and a total of 32,000 images were obtained, including: the original 1,000 images; the 3,000 images obtained when the different rotations were applied; 4,000 images with a watermark (applied to both the original and the rotated images); 4,000 images with a color mask; 4,000 images with a frame; 4,000 images with a watermark and a color mask; 4,000 images with a watermark and a frame; 4,000 images with a color mask and a frame; and 4,000 with a watermark, color mask and frame. This dataset was published in [38]. However, as previously mentioned, color mask has no influence over the results of hash-based algorithms, so those images have not been included in the final test dataset.

**Fig 8:**
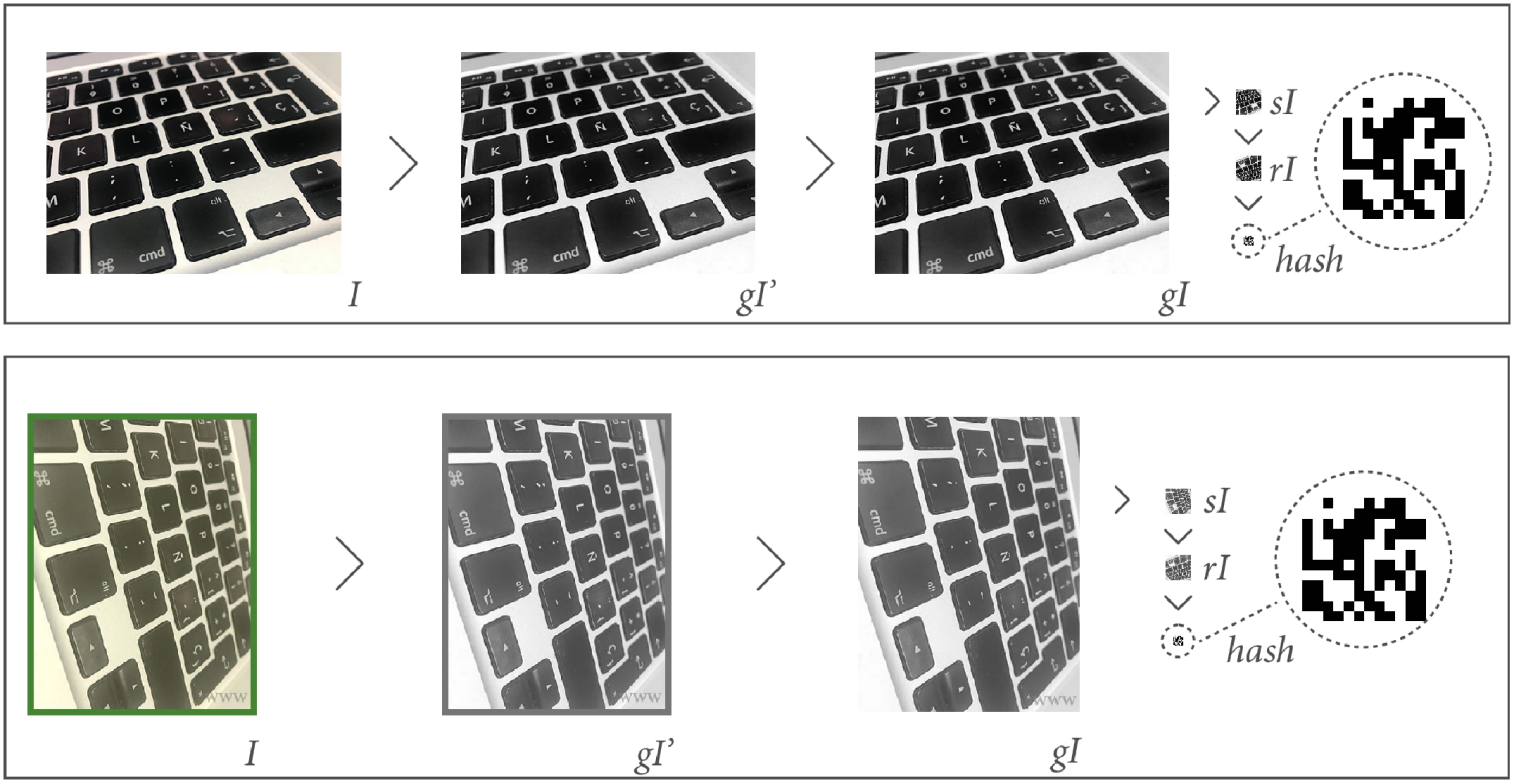
Example of the system process.

Fig 8 details an example of the processing of two images. On the top side, the processing of an image obtained directly from the original is shown, rescaled so that the longest side measures 450 pixels. On the bottom side, there is an image with a yellowish hue, rotated 90°, with an outer frame, and a watermark in the bottom-right corner. The result in both cases is a 144-digit value composed of 1 and 0, as detailed in Algorithm 3.

For the image on the left, the obtained hash is: 1101110100111111100000110101000 1100001000001000001100011011001100011110100001010010111100010001000111010101 000111001110000010100110011001010010. And the hash for the image on the right is: 1101110100111111100000110101000110000100000100000110001101100110001111010000 1010010111100010001000111010101000111001110000010100110011001010010. After calculating Hamming distance, the system determines that both images are 100% equal.

To evaluate the performance of the algorithm, it was compared with the different implementations of hash-based algorithms. 16,000 images were obtained from the total set of the images to which different transformations were applied. The success rate was evaluated by considering the result to be a success when the system associated the modified image with the original image of the dataset as the most similar, having a similarity value greater than 99%. The applied transformations and the images used are shown in Table 1.

**Table 1:** Test dataset.

	n	r	w	f	f,w	f,r	w,r	f,w,r	Total
**Images**	1k	3k	1k	1k	1k	3k	3k	3k	16k

Legend: n = none; r = rotation; w = watermark; f = frame

All of these images were provided as input using the implementations of the pHash, aHas, and dHash algorithms, the TinEye API and the proposed algorithm.

Following these indications, the results obtained are reflected in Table 2, where all images that were cataloged as equal, and indeed were, are considered a success.

**Table 2:** Hit rate for hash based algorithms.

	n	r	w	f	f,w	f,r	w,r	f,w,r	Avg.
pHash	**100%**	0%	**75%**	0%	0%	0%	0%	0%	11%
aHash	**100%**	0%	74%	0%	0%	0%	0%	0%	11%
dHash	**100%**	0%	**75%**	0%	0%	0%	0%	0%	11%
TinEye	**100%**	2%	**75%**	**100%**	0%	0%	0%	0%	18%
**Prop.**	**100%**	**100%**	**75%**	**100%**	**74%**	**90%**	**75%**	**73%**	**85%**

Legend: n = none; f = frame; w = watermark; r = rotation

It can be observed that the proposed system shows a better result in all transformations except when a watermark is included. In that case, TinEye, dHash and pHash show the same success rate.

Regarding the physical computational architecture, up to 40 nodes available with two cores with up to 2.4GHz and 4GB of RAM. The value of *rpn* depends on the characteristics of each node, such as the operative system, database, hardware and software, etc. Since we are using nodes with the same characteristics and the same configuration, we can assume these variables as constants. Another constant used is the longest side size of every image, 450 pixels. A variable that cannot be assumed as constant is the number of image hashes stored in the database, because its size affects the *rpn* significantly.

The search algorithm using hashing has a logarithmic order but if we assume it grows lineally we add security to the system (if we do not make this assumption it turns out to be more costly), as to that which has been considered lineal, we are guaranteed that the requests are dealt with in less than two seconds, on the cost overrun that linearization may imply. This way we can estimate a linear function based on the number of hashes stored in the database and calculate the *rpn*.

With the aim of calculating the number of requests per second, we carried out tests using our system. One test consists of asking the system to process a number *N* of images and obtain the time *T* that the system needs to process all of them and therefore, finding out the number of seconds needed per image *N*/*T*. To contrast these results, we process one image and calculate the time taken, in these trials we maintain the hash database size constant. Every node with these characteristics can serve up to 24 requests per second without exceeding the two-second restriction on processing.

## Conclusion and future work

The proposed system improves the current state of the art of image matching by including images which have been slightly modified by the inclusion of a watermark, outer borders, or rotations of 90°, 180°, and 270°.

The results are robust in terms of the insertion of edges and rotations. However, with the insertion of watermarks which have considerably altered the image, none of the algorithms was able to associate the images with precision. In fact, in order not to introduce false positives (identify images as equal images when they are in fact not), it is necessary to compromise the detail with which the analysis is performed.

As a future line of work, this solution will be incorporated into an existing job search social network in order to suggest contacts to users who have published or shared the same images. Regarding the image matching system, different solutions capable of associating images whose proportions have been modified by the user, either by trimming and removing part of the exterior of the image or by having deformed the image, are being evaluated. This evolution could make it possible to check any possible rotations within 360°.

Concerning the node forecasting aspect/feature, it could be improved by adding techniques capable of detecting DDoS (Distributed Denial of Service) attacks when receiving the requests. In this way, the processing of malicious requests will not occur and will not consume those computing resources.

Moreover, the hybrid system can be adjusted in different ways to achieve different levels in the security-saving balance by playing with the window and confidence interval of the dynamic system, as well as with the function that combines the result of the dynamic system with the static system result.

## References

[1] Kambatla K, Kollias G, Kumar V, Grama A. Trends in big data analytics. Journal of Parallel and Distributed Computing. 2014;74(7):2561–2573. doi: 10.1016/j.jpdc.2014.01.003

[2] beBee. beBee, Affinity Networking; 2017 [cited 2018 May 12]. Available from: https://www.bebee.com

[3] Rodríguez S, de Paz Y, Bajo J, Corchado JM. Social-based planning model for multiagent systems. Expert Systems with Applications. 2011;38(10):13005–13023. doi: 10.1016/j.eswa.2011.04.101

[4] Russell S, Norvig P, Intelligence A. A modern approach. Artificial Intelligence Prentice-Hall, Egnlewood Cliffs. 1995;25:27.

[5] Razavi R, Perrot JF, Guelfi N. Adaptive modeling: an approach and a method for implementing adaptive agents. In: International Workshop on Massively Multiagent Systems. Springer; 2004. p. 136–148.

[6] Argente E, Julian V, Botti V. Multi-agent system development based on organizations. Electronic Notes in Theoretical Computer Science. 2006;150(3):55–71. doi: 10.1016/j.entcs.2006.03.005

[7] Rodriguez S, Julián V, Bajo J, Carrascosa C, Botti V, Corchado JM. Agent-based virtual organization architecture. Engineering Applications of Artificial Intelligence. 2011;24(5):895–910. doi: 10.1016/j.engappai.2011.02.003

[8] Argente-Villaplana, E. GORMAS: Guías para el desarrollo de Sistemas Multiagente abiertos basados en organizaciones Engineering Applications of Artificial Intelligence. 2008.

[9] Erickson T, Kellogg WA. Social translucence: an approach to designing systems that support social processes. ACM transactions on computer-human interaction (TOCHI). 2000;7(1):59–83. doi: 10.1145/344949.345004

[10] Robertson D, Giunchiglia F. Programming the social computer. Philosophical Transactions of the Royal Society of London A: Mathematical, Physical and Engineering Sciences. 2013;371(1987):20120379 doi: 10.1098/rsta.2012.0379 10.1098/rsta.2012.0379 23419848

[11] Charron C, Favier J, Li C. Social computing. Forrester Research. 2006.

[12] Wang FY, Carley KM, Zeng D, Mao W. Social computing: From social informatics to social intelligence. IEEE Intelligent Systems. 2007;22(2). doi: 10.1109/MIS.2007.41

[13] Heras S, Jordán J, Botti V, Julián V. Argue to agree: A case-based argumentation approach. International Journal of Approximate Reasoning. 2013;54(1):82–108. doi: 10.1016/j.ijar.2012.06.005

[14] Foster I, Kesselman C, Tuecke S. The anatomy of the grid: Enabling scalable virtual organizations. International journal of high performance computing applications. 2001;15(3):200–222. doi: 10.1177/109434200101500302

[15] Garcia-Fornes A, Hübner JF, Omicini A, Rodriguez-Aguilar JA, Botti V. Infrastructures and tools for multiagent systems for the new generation of distributed systems. Engineering Applications of Artificial Intelligence. 2011;24(7):1095–1097. doi: 10.1016/j.engappai.2011.06.012

[16] Coria JAG, Castellanos-Garzón JA, Corchado JM. Intelligent business processes composition based on multi-agent systems. Expert Systems with Applications. 2014;41(4):1189–1205. doi: 10.1016/j.eswa.2013.08.003

[17] Heras S, De la Prieta F, Julian V, Rodríguez S, Botti V, Bajo J, Corchado JM. Agreement technologies and their use in cloud computing environments Progress in Artificial Intelligence. 2012;1(4):277–290. doi: 10.1007/s13748-012-0031-9

[18] Smith SM, Brady JM. SUSAN—A new approach to low level image processing. International journal of computer vision. 1997;23(1):45–78. doi: 10.1023/A:1007963824710

[19] Reitmayr G, Drummond T. Going out: robust model-based tracking for outdoor augmented reality. In: Proceedings of the 5th IEEE and ACM International Symposium on Mixed and Augmented Reality. IEEE Computer Society; 2006. p. 109–118.

[20] Bay H, Tuytelaars T, Van Gool L. Surf: Speeded up robust features. In: European conference on computer vision. Springer; 2006. p. 404–417.

[21] Rublee E, Rabaud V, Konolige K, Bradski G. ORB: An efficient alternative to SIFT or SURF. In: Computer Vision (ICCV), 2011 IEEE International Conference on. IEEE; 2011. p. 2564–2571.

[22] Matas J, Chum O, Urban M, Pajdla T. Robust wide-baseline stereo from maximally stable extremal regions. Image and vision computing. 2004;22(10):761–767. doi: 10.1016/j.imavis.2004.02.006

[23] Deng H, Zhang W, Mortensen E, Dietterich T, Shapiro L. Principal curvature-based region detector for object recognition. In: Computer Vision and Pattern Recognition, 2007. CVPR’07. IEEE Conference on. IEEE; 2007. p. 1–8.

[24] Norouzi M, Fleet DJ, Salakhutdinov RR. Hamming distance metric learning. In: Advances in neural information processing systems; 2012. p. 1061–1069.

[25] Krawetz N. Looks Like It; 2011 [cited 2018 May 12]. In: The Hacker Factor Blog [Internet]. Available from: http://www.hackerfactor.com/blog/index.php?/archives/432-Looks-LikeIt.html

[26] Krawetz N. Kind of Like That; 2013 [cited 2018 May 12]. In: The Hacker Factor Blog [Internet]. Available from: http://www.hackerfactor.com/blog/?/archives/529-Kind-of-Like-That.html

[27] Aghav S, Kumar A, Gadakar G, Mehta A, Mhaisane A. Mitigation of rotational constraints in image based plagiarism detection using perceptual hash. Int J Comput Sci Trends Technol. 2014;2:28–32.

[28] TinEye. TinEye Reverse Image Search; 2017 [cited 2018 May 12]. Available from: https://www.tineye.com/

[29] Chambers JC, Mullick SK, Smith DD. How to Choose right Forecasting Technique. Harvard business review. 1971;49(4):45.

[30] Mahmud T, Hasan M, Chakraborty A, Roy-Chowdhury AK. A poisson process model for activity forecasting. In: Image Processing (ICIP), 2016 IEEE International Conference on. IEEE; 2016. p. 3339–3343.

[31] Weinberg J, Brown LD, Stroud JR. Bayesian forecasting of an inhomogeneous Poisson process with applications to call center data. Journal of the American Statistical Association. 2007;102(480):1185–1198. doi: 10.1198/016214506000001455

[32] Hong WC, Dong Y, Chen LY, Wei SY. SVR with hybrid chaotic genetic algorithms for tourism demand forecasting. Applied Soft Computing. 2011;11(2):1881–1890. doi: 10.1016/j.asoc.2010.06.003

[33] Barrow D, Kourentzes N. The impact of special days in call arrivals forecasting: a neural network approach to modelling special days. European Journal of Operational Research. 2016.

[34] Rao KR, Yip P. Discrete cosine transform: algorithms, advantages, applications. Academic press; 2014.

[35] Burkhard WA, Keller RM. Some approaches to best-match file searching. Communications of the ACM. 1973;16(4):230–236. doi: 10.1145/362003.362025

[36] Pixabay. Free Images—Pixabay; 2017 [cited 2018 May 12]. Available from: https://www.pixabay.com/

[37] Rivas A, Chamoso P. Image dataset; 2017 [cited 2018 May 12]. Database: figshare [Internet]. Available from: https://doi.org/10.6084/m9.figshare.5729064

[38] Rivas A, Chamoso P. Image dataset; 2017 [cited 2018 May 12]. Database: figshare [Internet]. Available from: https://doi.org/10.6084/m9.figshare.5692723

